# ΔNp73 regulates the expression of the multidrug-resistance genes *ABCB1* and *ABCB5* in breast cancer and melanoma cells - a short report

**DOI:** 10.1007/s13402-017-0340-x

**Published:** 2017-07-04

**Authors:** Habib A. M. Sakil, Marina Stantic, Johanna Wolfsberger, Suzanne Egyhazi Brage, Johan Hansson, Margareta T. Wilhelm

**Affiliations:** 1 Department of Microbiology, Tumor and Cell biology (MTC), Karolinska Institutet, 171 77 Stockholm, Sweden; 2 Department of Oncology-Pathology, Karolinska Institutet, 171 76 Stockholm, Sweden

**Keywords:** p73, Multi-drug resistance genes, ABC transporters, Breast cancer, Melanoma, p53 family

## Abstract

**Purpose:**

Multidrug resistance (MDR) is a major cause of treatment failure. In cancer cells, MDR is often caused by an increased efflux of therapeutic drugs mediated by an up-regulation of ATP binding cassette (ABC) transporters. It has previously been shown that oncogenic ΔNp73 plays an important role in chemo-resistance. Here we aimed at unraveling the role of ΔNp73 in regulating multidrug resistance in breast cancer and melanoma cells.

**Methods:**

KEGG pathway analysis was used to identify pathways enriched in breast cancer samples with a high ΔNp73 expression. We found that the ABC transporter pathway was most enriched. The expression of selected ABC transporters was analyzed using qRT-PCR upon siRNA/shRNA-mediated knockdown or exogenous overexpression of ΔNp73 in the breast cancer-derived cell lines MCF7 and MDA-MB-231, as well as in primary melanoma samples and in the melanoma-derived cell line SK-MEL-28. The ability to efflux doxorubicin and the concomitant effects on cell proliferation were assessed using flow cytometry and WST-1 assays.

**Results:**

We found that high ΔNp73 levels correlate with a general up-regulation of ABC transporters in breast cancer samples. In addition, we found that exogenous expression of ΔNp73 led to an increase in the expression of ABCB1 and ABCB5 in the breast cancer-derived cell lines tested, while knocking down of ΔNp73 resulted in a reduction in ABCB1 and ABCB5 expression. In addition, we found that ΔNp73 reduction leads to an intracellular retention of doxorubicin in MDA-MB-231 and MCF7 cells and a concomitant decrease in cell proliferation. The effect of ΔNp73 on ABCB5 expression was further confirmed in metastases from melanoma patients and in the melanoma-derived cell line SK-MEL-28.

**Conclusions:**

Our data support a role for ΔNp73 in the multidrug-resistance of breast cancer and melanoma cells.

**Electronic supplementary material:**

The online version of this article (doi:10.1007/s13402-017-0340-x) contains supplementary material, which is available to authorized users.

## Introduction

ATP binding cassette (ABC) transmembrane proteins are commonly expressed in both eukaryotes and prokaryotes. In eukaryotes, they mainly function as exporters, whereas in prokaryotes they act as both exporters and importers [1]. In humans, the ABC superfamily comprises 49 ABC genes grouped into 7 subfamilies represented as A to G based on structure and sequence homology. As active transporter and efflux pumps, ABC proteins utilize ATP-mediated energy for exporting sugars, lipids, small ions, peptides and drug molecules through extracellular and intracellular membranes [2].

High ABC expression is a common feature of a variety of human cancers and, due to their drug export capacity, a hurdle for successful treatment. Among the most well-studied human ABC transporters is the ABC subfamily B member 1 (ABCB1/MDR1/P-glycoprotein), which was first identified as a multidrug resistance protein after discovering that cells expressing ABCB1 acquired resistance to amphiphilic drugs [3]. A high expression of ABCB1 has been associated with a poor prognosis in various cancers, including hepatic carcinoma, colon carcinoma, kidney cancer, osteosarcoma, soft tissue sarcoma and hematological malignancies, including leukemia and lymphoma [4–6]. Furthermore, it has been shown that melanoma, breast and ovarian cancer cells depend on ABCB1 for developing multidrug resistance (MDR) [7–9]. As a drug transporter, ABCB1 can efflux different types of hydrophobic molecules, including doxorubicin, colchicine, adriamycin, vinblastine and taxane drugs [10]. Other ABC transporters that have been shown to confer chemo-resistance to cells include ABCG2 (Breast Cancer Resistance Protein/BCRP), ABCC1 (Multidrug Resistance Protein 1/MRP1) and ABCB5. ABCB5 was first identified as a regulator of membrane potential and cellular fusion [11] and has also been shown to serve as a marker for limbal stem cells, skin progenitor cells and melanoma stem cells, suggesting a role in maintaining stemness [12–14]. Similar to ABCB1, ABCB5 increases the efflux of drug molecules from cancer cells and, by doing so, promotes their chemo-resistance [13]. In addition, ABCB5 has been found to be involved in self-renewal, differentiation and melanoma progression [12]. Drug-resistant metastatic melanoma cells tend to be ABCB5 enriched and may show tumor re-growth after dacarbazine, temozolomide or vemurafenib treatment [15].

The tumor suppressor p53 has been shown to downregulate ABCB1 via binding to its promoter and, thus, to decrease its expression [16], while mutant p53 has been shown to promote the expression of ABCB1 and, thus, to cause chemo-resistance in colon cancer [17]. The p73 gene, another member of the p53-family, encodes several isoforms with different biological functions. Isoforms expressing the transactivation (TA) domain, TAp73-isoforms, act similar to p53 and induce cell cycle arrest and apoptosis in response to cellular stress. In addition, the p73 gene encodes N-terminally truncated isoforms, either through transcription from an internal promoter (P2; ΔNp73) or through alternative splicing of N-terminal exons (p73ΔEx2, p73ΔEx2/3 and ΔN′p73), collectively called ΔNp73 isoforms [18]. While TAp73 is considered to act as a tumor suppressor, the ΔNp73 isoforms induce oncogenic properties inducing anchorage independent growth, cell proliferation and tumor angiogenesis [19–21]. Furthermore, high levels of ΔNp73 have been correlated with a poor clinical outcome in neuroblastoma, medulloblastoma, lung, ovarian, prostate, colon and breast cancer patients [18]. We have previously reported that ΔNp73^−/−^ mouse embryonic fibroblasts are sensitized towards cytotoxic drugs [22]. Moreover, high ΔNp73 levels have been correlated with a poor drug response in several types of cancer, suggesting that ΔNp73 confers drug resistance [18]. Here, we report that ABC transporter pathways are among the most enriched biological pathways in breast cancer patients with a high expression of ΔNp73. Furthermore, we show that ΔNp73 enhances ABCB1 and ABCB5 expression in both p53 wild-type and p53 mutant breast cancer cells. Knockdown of ΔNp73 was found to reduce ABCB1 and ABCB5 expression and to decrease doxorubicin export and cell proliferation. In addition, we found that expression of the ΔNp73 isoform p73ΔEx2/3 correlates with ABCB5 expression in metastases from melanoma patients and in melanoma-derived cells.

## Material and methods

### Cell culture and patient samples

The human breast cancer-derived cell lines MCF7 and MDA-MB-231 were verified by STR profiling at the ECACC, UK. The human melanoma-derived cell line SK-MEL-28 was purchased from the ATCC. MCF7, MDA-MB-231 and HEK293T cells were maintained in DMEM medium supplemented with 10% FBS, 100 U/ml penicillin and 100 mg/ml streptomycin (Hyclone, GE-Healthcare). SK-MEL-28 cells were maintained in MEM medium (Gibco, Thermo Fisher Scientific) supplemented with 10% FBS, 2 mM L-glutamine, 100 U/ml penicillin and 100 mg/ml streptomycin (Hyclone, GE-Healthcare). For patient samples, total RNA from cutaneous melanoma metastases was extracted from 37 patients who underwent surgery at the Karolinska University Hospital, Stockholm, Sweden. The specimens were fresh frozen in liquid nitrogen and kept in a biobank until use. The biobanking and analysis of patient samples were approved by the Stockholm Regional Ethics Committee.

### Transfection and cloning assays

MCF7, MDA-MB-231 and SK-MEL-28 cells were seeded in 6-well plates at densities of 350,000, 700,000 and 350,000 cells/well, respectively. 24 h after seeding, the cells were transfected with 1 μg pcDNA3.1 (control), pcDNA-ΔNp73α, pcDNA-p73ΔEx2/3α or pcDNA-p73ΔEx2/3β using Lipofectamine 3000 according to the manufacturer’s instructions (Thermo Fisher Scientific) and harvested for RNA and protein extraction 16 h after transfection. p73ΔEx2/3α and p73ΔEx2/3β were amplified from melanoma patient cDNA using Phusion High Fidelity DNA Polymerase (New England Biolabs) and cloning primers p73ΔEx2/3-F: 5′-ACGGATCCATGGACCAGATGAGCAGCCGC, and p73ΔEx2/3α-R: 5′-ACGATATCTCAGTGGATCTCGGCCTCC, or p73ΔEx2/3β-R: 5′-ACGATATCTCAGGGCCCCCAGGTCCTGACGAG. The resulting amplified fragments were cloned into a pcDNA3.1 expression vector. MCF7 and MDA-MB-231 cells with stable ΔNp73 knockdown were generated by lentiviral transduction of a short hairpin RNA directed against ΔNp73. The lentivirus was constructed by cloning oligo 5′-CCGGGACAGAACTAAGGGAGATGTTCAAGAGACATCTCCCTTAGTTCTGTCTTTTTG-3′ into a pLKO.1-puro vector. Lentivirus was produced in HEK293T cells using the packaging and envelope constructs pCMVΔ8.2 and pMD.G-VSV-G as described by Szulc et al. [23] (pLKO.1-puro, pCMVΔ8.2 and pMD.G-VSV-G were gifts from Bob Weinberg; Addgene plasmids #8453, #8454 and #8455) [24]). For siRNA transfection, we used a control siRNA and ΔNp73-siRNA oligo 5′- GCGCCUACCAUGCUGUACGUC[dT][dT] (Sigma-Aldrich) with Lipofectamine RNAiMAX according to the manufacturer’s instructions (Thermo Fisher Scientific) and harvested the cells for analysis 48 h (qRT-PCR) or 72 h (efflux assay) after transfection.

### cDNA synthesis and quantitative real-time PCR

Total RNA was isolated using a Direct-zol™ RNA extraction kit (Zymoresearch) according to the manufacturer’s instructions. 500 ng total RNA was used for cDNA synthesis (iScript cDNA synthesis kit, Bio-Rad) and quantitative real-time PCR (qRT-PCR) was performed using an ABI StepOnePlus system (Applied Biosystems) in conjunction with a Taqman master mix (Thermo Fisher Scientific) or a SybrGreen mix (iTaq universal SybrGreen mix, Bio-Rad). Each sample was run in triplicate and normalized to GAPDH or 28S RNA. Gene expression levels were analyzed using the ΔΔCT method and shown as relative fold changes. Primer sequences are listed in Supplementary Table 1.

### Western blotting

Protein lysates were prepared using RIPA buffer (Sigma-Aldrich) containing 1× protease inhibitor (Thermo Fisher Scientific). Twenty μg of total protein was fractionated using 10% Bolt Bis-Tris gels (Thermo Fisher Scientific) and transferred to nitrocellulose membranes using Trans-Blot® Turbo™ Transfer System (Bio-Rad). The antibodies used are anti-p73 (ab17230, 1:2000, Abcam) and anti-β-actin (Ab49900, 1:10,000, Abcam). Bands were detected using ECL reagent (Amersham) and a chemiDoc™ XRS+ imaging system (Bio-Rad).

### Cell proliferation assay

The seeding densities used were 3000 cells/well (MCF7-shCtrl and MCF7-shΔNp73) and 4000 cells/well (MDA-MB-231-shCtrl and MDA-MB-231-shΔNp73) in triplicates in 96 well plates. The respective cells were treated with doxorubicin (0 μM, 0.125 μM, 0.25 μM, 0.5 μM and 1 μM) for 48 h after which cell proliferation was assessed using WST-1 reagent (Roche) according to the manufacturer’s instructions. Cell viability was normalised to a DMSO control. The proliferation rate of the DMSO control was set at 100%.

### Efflux assay

Cells were treated with 1 μM doxorubicin for 30 min followed by immediate harvesting, or allowed to recover in fresh medium for 3 h before harvesting. Next, intracellular doxorubicin was measured by fluorescence intensity using flow cytometry (FACS Calibur). The data were analyzed using FlowJo software.

### Statistical analysis

Statistical analyses were performed using Student’s t-test in GraphPad Prism 6 (GraphPad Software, USA). Correlations between gene expression levels in human melanomas were evaluated using Pearson’s correlation test. All experiments were performed at least three times independent of each other unless stated otherwise, and data are presented as mean ± SEM. For all statistical analyses *p* < 0.05 was considered significant.

## Results and discussion

**Fig. 1:**
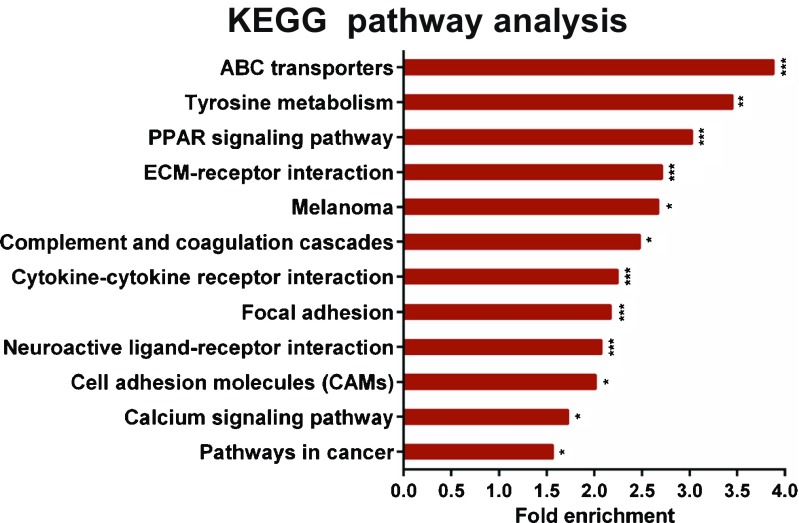
KEGG pathway enrichment analysis. Selected pathways (*p* < 0.05) enriched in breast cancer patients with a high ΔNp73 expression. *p < 0.05, **p < 0.01 and ***p < 0.005

We have previously reported, using publically available expression data from The Cancer Genome Atlas (TCGA), that human breast cancer samples with a high ΔNp73 expression show enrichment in angiogenesis and hypoxia pathways [20]. To identify other biological pathways that are upregulated in ΔNp73 expressing breast cancer samples we filtered the dataset we previously published [20] on genes with a significant (≥ 2-fold) upregulation. The resulting 916 upregulated genes (Supplementary Table 2) were subjected to Kyoto Encyclopedia of Genes and Genomes (KEGG) pathway analysis using the Database for Annotation, Visualization and Integrated Discovery (DAVID) bioinformatics online resource (http://david.abcc.ncifcrf.gov/) version 6.7 [25]. Among the upregulated KEGG pathways, the ABC transporters were found to be most enriched. Other significantly upregulated pathways included the Tyrosine metabolism, Extra cellular matrix-receptor interactions, Cell adhesion and Focal adhesion pathways (Fig. 1, Supplementary Table 3). We decided to focus our study on the ABC transporters considering the important role they play in MDR. Among the ABC transporter genes upregulated in the ΔNp73-expressing samples, we found that the ABC subfamily A (ABCA5, ABCA6, ABCA8, ABCA9, ABCA10), ABC subfamily B (ABCB1 and ABCB5), ABC subfamily D (ABCD2) and ABC subfamily G (ABCG2) were significantly upregulated (Table 1). ABCA5 has previously been reported to be upregulated by ΔNp73-isoforms in malignant melanoma [26]. Additionally, a correlation between ABCB1 and p73 isoforms has been found in gastric cancer, colon cancer and neuroblastoma [27–29]. To the best of our knowledge this is the first time that the relationship between ABC transporters and ΔNp73 isoforms is investigated in breast cancer. Our data not only confirm previous reports on other types of malignancies, but also include more ABC transporters, suggesting that ΔNp73 augments several types of drug efflux pathways.

**Table 1:** Significantly upregulated ABC transporter genes in ΔNp73 high-expressing versus ΔNp73 non-expressing breast cancer samples

Gene name	Fold change	*P* value
ABCB5	5,20	7E-22
ABCA10	4,72	3E-25
ABCA9	3,52	2E-21
ABCB1	3,33	1E-24
ABCA8	3,27	6E-20
ABCA6	2,65	5E-18
ABCD2	2,51	5E-12
ABCG2	2,13	5E-17
ABCA5	2,04	1E-18

**Fig. 2:**
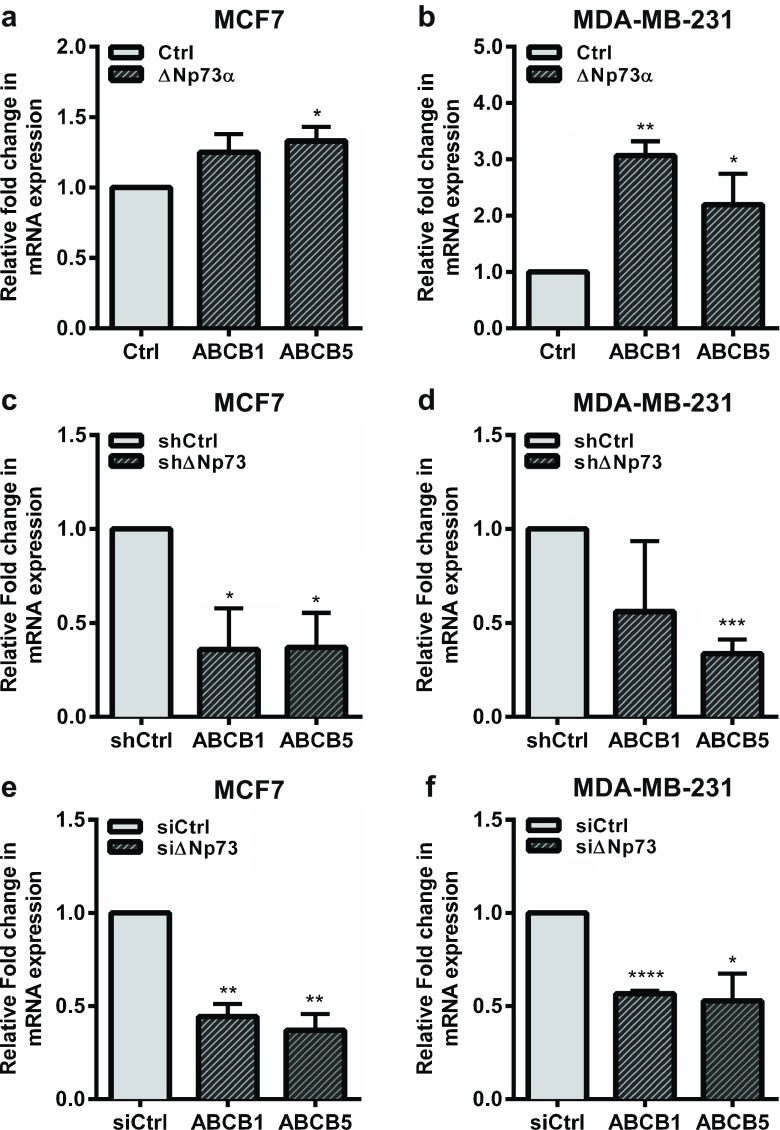
ΔNp73 upregulates ABCB1 and ABCB5 expression in human breast cancer cells. mRNA expression analysis of ABC genes using qRT-PCR. (**a**, **b**) Exogenous expression of ΔNp73α in MCF7 and MDA-MB-231 cells upregulates ABCB1 and ABCB5 mRNA expression levels. (**c**, **d**) shRNA and (**e**, **f**) siRNA-mediated knockdown of ΔNp73 in MCF7 and MDA-MB-231 cells results in downregulation of ABCB1 and ABCB5 mRNA expression levels. All samples were run in triplicate in three independent experiments and normalized to 28S mRNA. Relative expression was calculated using the ΔΔCT method, and presented as mean fold change ± S.E.M. *p < 0.05, **p < 0.01 and ***p < 0.005

To validate the effect of ΔNp73 expression on the various ABC transporters, we exogenously expressed ΔNp73 in two human breast adenocarcinoma-derived cell lines, MCF7 and MDA-MB-231. Using qRT-PCR we assessed the expression of the top five upregulated ABC transporter genes listed in Table 1, i.e., ABCB5, ABCA10, ABCA9, ABCB1 and ABCA8. Since we could not detect any expression of ABCA8, ABCA9 or ABCB10 in either MCF7 or MDA-MB-231 cells, we focused our study on ABCB1 and ABCB5. In response to the exogenous over-expression of ΔNp73α we found that ABCB5 was significantly upregulated in MCF7 and MDA-MB-231 cells, and that ABCB1 was significantly upregulated in MDA-MB-231 cells (Fig. 2a and b, Supplementary Fig. 1). Interestingly, ABCB1 has previously been reported to be upregulated by ΔNp73 in gastric cancer cells through blocking p53-mediated repression of the *ABCB1* gene promoter [29]. Here, we found that ΔNp73 can enhance ABCB1 expression in mutant p53 (p53R280K) MDA-MB-231 cells, suggesting that ΔNp73 can also increase ABCB1 expression in a p53-independent manner.

To confirm the effect of ΔNp73 on ABCB1 and ABCB5 expression, we generated MCF7 and MDA-MB-231 cells stably expressing a shRNA directed against ΔNp73. We found that the shRNA-mediated ΔNp73 knockdown significantly downregulated ABCB1 expression in MCF7 cells and ABCB5 expression in both MCF7 and MDA-MB-231 cells (Fig. 2c and d, Supplementary Fig. 2a and b). To exclude any off-target effects of the shRNA and to obtain more efficient knockdown, we designed a siRNA targeting a different part of the ΔNp73 mRNA and transiently transfected it into MCF7 and MDA-MB-231 cells. Again, we observed a significant downregulation of ABCB1 and ABCB5 in both cell lines tested (Fig. 2e and f, Supplementary Fig. 2c and d). Taken together, our data suggest that ΔNp73 positively regulates the expression of ABCB1 and ABCB5 in breast cancer cells.

**Fig. 3:**
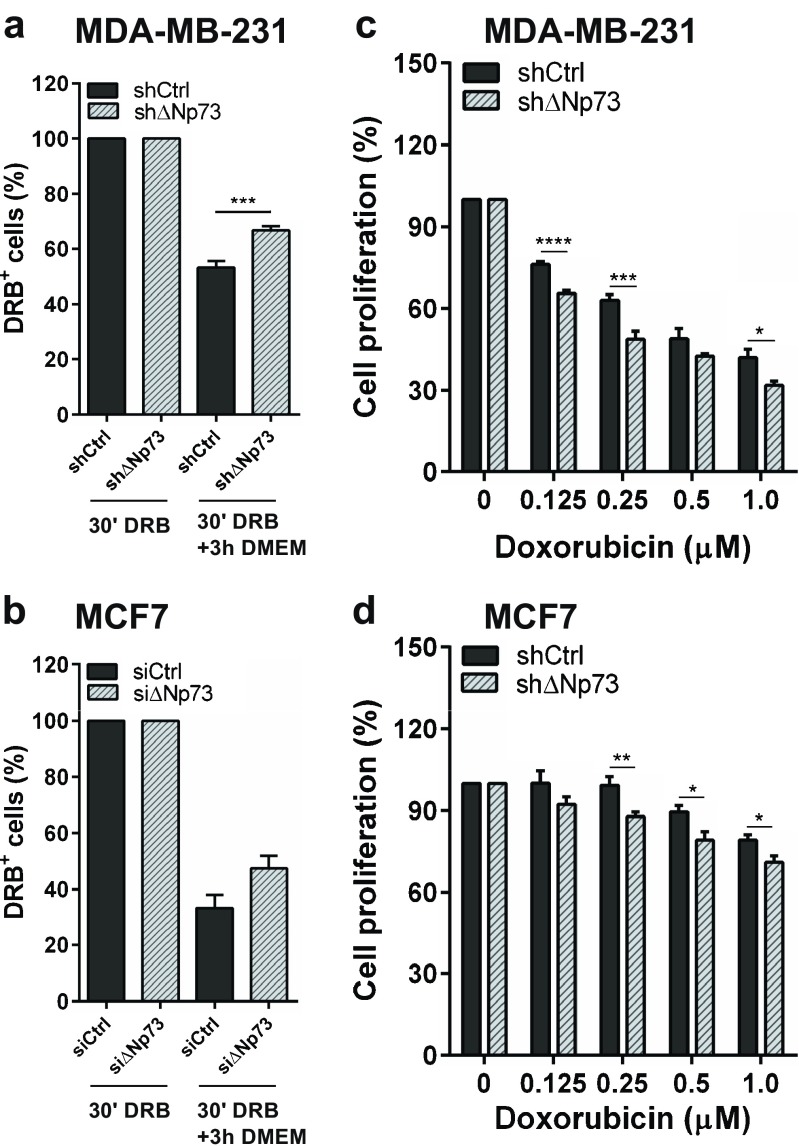
Knockdown of ΔNp73 decreases doxorubicin efflux and cell proliferation. (**a**, **b)** shΔNp73 MDA-MB-231 and siΔNp73 MCF7 cells show increased retention of doxorubicin (DRB) compared to control cells after 30 min DRB incubation followed by 3 h incubation in normal media. (**c**, **d**) Significant reduction of cell proliferation in shΔNp73 MDA-MB-231 and MCF7 cells upon doxorubicin treatment as measured by WST-1 activity. All samples were run in triplicate in three independent experiments. Data are presented as mean ± SEM. *p < 0.05, **p < 0.01, ***p < 0.005 and ****p < 0.001

Previously, the drug efflux capacity of ABCB1 and ABCB5 has been shown to enhance resistance to doxorubicin in breast cancer cells [30, 31]. Interestingly, high ΔNp73 levels have been linked to limited therapeutic responses in various cancer types, highlighting a role for ΔNp73 in drug resistance [18]. To test whether knocking down ΔNp73 affects the intracellular accumulation of doxorubicin, we analyzed cells incubated with doxorubicin using flow cytometry. By doing so, we could not detect any significant change in cellular uptake between shControl MDA-MB-231 cells and shΔNp73 MDA-MB-231 cells after 30 min incubation in media containing 1 μM doxorubicin, suggesting that the influx of doxorubicin is not affected. However, we did observe significantly higher levels of intracellular doxorubicin in shΔNp73 MDA-MB-231 cells following a three hour incubation in normal medium (Fig. 3a), suggesting that shΔNp73 cells retain doxorubicin and that the efflux capacity is higher in shControl cells than in shΔNp73 cells (Fig. 3a). Similar results were obtained for siΔNp73 MCF7 cells compared to siControl MCF7 cells (Fig. 3b). These effects were accompanied by a decrease in proliferation of both shΔNp73 MDA-MB-231 and shΔNp73 MCF7 cells treated with doxorubicin (Fig. 3c and d).

**Fig. 4:**
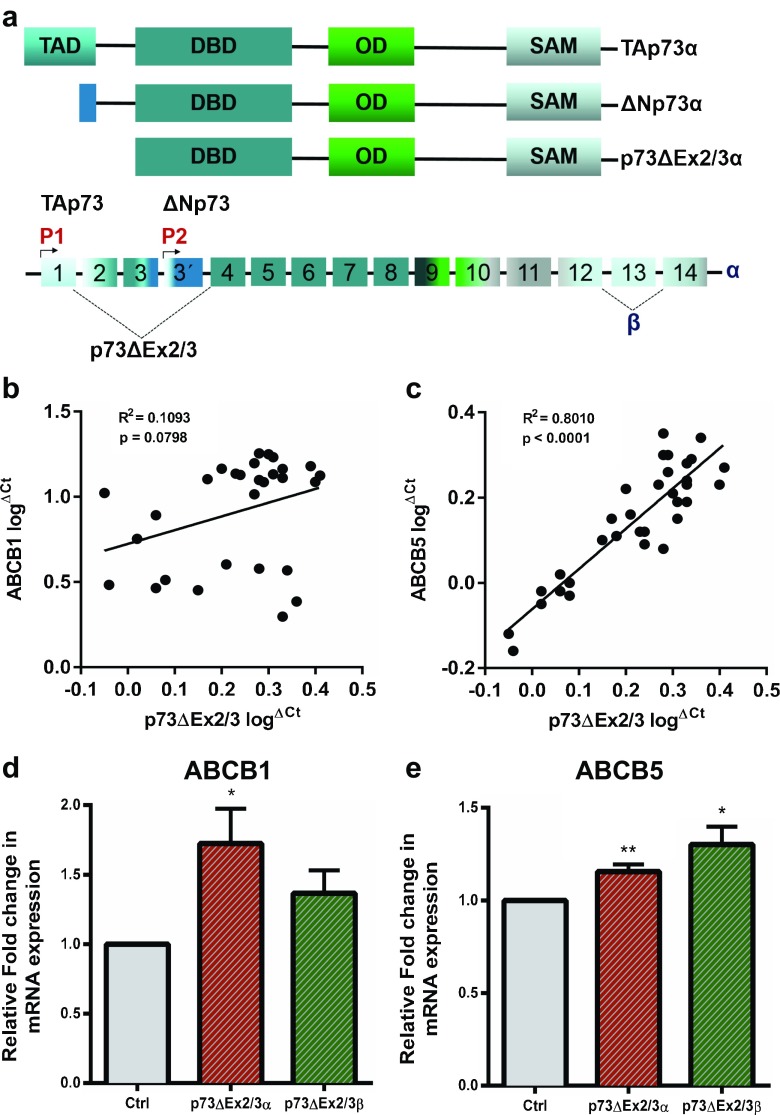
p73ΔEx2/3 expression correlates with ABCB5 expression in metastatic melanoma tumors. **a** Schematic representation of the *TP73* gene structure. The P1 and P2 promoters give rise to two different classes of isoforms, TAp73 and ΔNp73, respectively. Alternate splicing of N-terminal exons produces the p73ΔEx2/3 isoforms. C-terminal splicing generates additional isoforms. **b**, **c** qRT-PCR analysis reveals a statistically significant correlation between ABCB5 and p73ΔEx2/3 expression (*n* = 33, *p* < 0.0001), whereas ABCB1 shows a weak correlation (*n* = 29, *p* = 0.0798). Each tumor sample was run in triplicate and mean log^ΔCt^ values were normalized to GAPDH and plotted. **d**, **e** ABCB1 and ABCB5 mRNA expression was analyzed upon overexpression of p73ΔEx2/3α and p73ΔEx2/3β in SK-MEL-28 cells. All samples were run in triplicate in three independent experiments. Data are presented as mean fold change ± SEM. *p < 0.05, **p < 0.01

Next, we set out to investigate whether ΔNp73 may have the same effect on ABCB1 and ABCB5 expression in another type of cancer. Considering the important role that ABCB5 plays as a driver in melanoma growth, aggressiveness and drug resistance [12, 13, 32], we first analyzed the correlation between ΔNp73, ABCB1 and ABCB5 expression in metastatic human melanoma samples. In melanoma, p73ΔEx2/3 is the predominantly expressed ΔNp73 isoform, with a similar activity as ΔNp73 [33]. p73ΔEx2/3 lacks the TA-domain as a result of alternative splicing (Fig. 4a). Additional splicing at the C-terminus may give rise to more splice variants, designated α–η (α and β variants are shown in Fig. 4a). Indeed, using qRT-PCR we readily detected p73ΔEx2/3 expression, but no P2-derived ΔNp73 expression, in melanoma patient samples, thereby confirming previous reports [33]. Interestingly, we found that p73ΔEx2/3 expression positively correlates with the expression of ABCB5 in primary human melanoma patient samples (Fig. 4c). In addition, we could observe a correlation between p73ΔEx2/3 and ABCB1 expression, although not statistically significant (*p* = 0.0798) (Fig. 4b). To further address the role of p73ΔEx2/3 in ABCB1 and ABCB5 expression, we cloned the p73ΔEx2/3α and p73ΔEx2/3β isoforms from the patient sample with the highest expression of endogenous p73ΔEx2/3, and exogenously expressed them in SK-MEL-28 melanoma-derived cells. Subsequently, we found a statistically significant upregulation of ABCB1 expression by p73ΔEx2/3α and of ABCB5 expression by both p73ΔEx2/3α and p73ΔEx2/3β (Fig. 4d and e). Taken together, we conclude that our data indicate that ΔNp73 regulates the expression of the MDR genes ABCB1 and ABCB5 and, by doing so, influences drug efflux mediated chemo-resistance in breast cancer cells. In accordance with these results, we observed a correlation between ΔNp73 and ABCB1 and ABCB5 expression in primary melanoma patient samples and confirmed a similar ΔNp73-driven impact on these MDR genes in a melanoma-derived cell line. Our data point at a possible molecular mechanism underlying the role of ΔNp73 in the acquisition of multi-drug resistance.

## Electronic supplementary material

ESM 1 (PDF 212 kb)

ESM 2 (PDF 105 kb)

ESM 3 (PDF 176 kb)

ESM 4 (PDF 570 kb)

ESM 5 (PDF 655 kb)
