# Supplementary material for: ΔNp73 regulates the expression of the multidrug-resistance genes ABCB1 and ABCB5 in breast cancer and melanoma cells - a short report
Source: Cell Oncol (Dordr). 2017 Jul 4;40(6):631–8. doi: 10.1007/s13402-017-0340-x (PMC5705756; doi:10.1007/s13402-017-0340-x)
Supplement: Supplementary file 1 — (PDF 212 kb) [file 13402_2017_340_MOESM1_ESM.pdf]

Article Title: ΔNp73 regulates the expression of the multidrug-resistance genes *ABCB1* and *ABCB5* in breast cancer and melanoma cells - a short report

Journal Name: Cellular Oncology

Authors Names: Habib A. M. Sakil1, Marina Stantic1, Johanna Wolfsberger1, Suzanne Egyhazi Brage2, Johan Hansson2, and Margareta T. Wilhelm1*

Affiliation: 1) Karolinska Institutet, Department of Microbiology, Tumor and Cell biology (MTC), 171 77 Stockholm, Sweden
2) Karolinska Institutet, Department of Oncology-Pathology, 171 76 Stockholm, Sweden

*Corresponding Author: Margareta T. Wilhelm
email: Margareta.Wilhelm@ki.se

Supplementary Table 1 Primer sequences used for qRT-PCR.

**Primer**	**Forward 5'-3'**	**Reverse 5'-3'**
ABCB5	ATTGGAGTGGTTAGTCAAGAGCC	AGTCACATCATCTCGTCCATACT
ABCA10	GGAGACGACAAATCTATGCAGTG	CCCAACAATGAGTTTCACGAGT
ABCA9	TTGCACCTGAATCCAAAACTACC	TGACTCTCACTGCGTCTATTGAA
ABCB1	TTGCTGCTTACATTCAGGTTTCA	AGCCTATCTCCTGTCGCATTA
ABCA8	TGAAATGGATGCCGATCCTTC	AGTATTGCAGTGATTTGGCCTT
p73ΔEx2/3	TGCAGGCCCAGTTCAATCTGC	TCGGTGTTGGAGGGGATGACA
28s	TTGAAAATCCGGGGGAG	ACATTGTTCCAACATGCC

**Taqman Primer**	
GAPDH	Cat # 4333764F, Thermo fisher scientific
ΔNp73	Cat # Hs01065727_m1, Thermo fisher scientific
