# Supplementary material for: ΔNp73 regulates the expression of the multidrug-resistance genes ABCB1 and ABCB5 in breast cancer and melanoma cells - a short report
Source: Cell Oncol (Dordr). 2017 Jul 4;40(6):631–8. doi: 10.1007/s13402-017-0340-x (PMC5705756; doi:10.1007/s13402-017-0340-x)
Supplement: Supplementary file 2 — (PDF 105 kb) [file 13402_2017_340_MOESM2_ESM.pdf]

Article Title: ΔNp73 regulates the expression of the multidrug-resistance genes *ABCB1* and *ABCB5* in breast cancer and melanoma cells - a short report
Journal Name: Cellular Oncology
Authors Names: Habib A. M. Sakil1, Marina Stantic1, Johanna Wolfsberger1, Suzanne Egyhazi Brage2, Johan Hansson2, and Margareta T. Wilhelm1*

Affiliation: 1) Karolinska Institutet, Department of Microbiology, Tumor and Cell biology (MTC), 171 77 Stockholm, Sweden
2) Karolinska Institutet, Department of Oncology-Pathology, 171 76 Stockholm, Sweden

*Corresponding Author: Margareta T. Wilhelm
email: Margareta.Wilhelm@ki.se

Supplementary Table 2 List of genes that were upregulated (>2 FC) in the ΔNp73 high expressing breast cancer patients. Adapted from Stantic et al. (2015) Proc Natl Acad Sci USA, 2015.112(1):p. 220-5.

GeneName	Fold Change	P.Val
PAK7	6,925	9E-37
C8orf79	3,392	1E-35
OVCH2	6,768	2E-35
GPR144	8,481	5E-35
B3GALT1	5,195	1E-34
NCRNA00086	3,782	1E-34
CAPZA3	3,652	1E-34
PIK3C2G	5,442	2E-33
DLK2	4,845	7E-33
C2orf40	6,320	2E-32
RNF186	5,409	2E-32
FAM196B	7,362	3E-32
SAMD5	5,692	4E-32
D4S234E	4,086	5E-32
LPPR1	4,986	8E-32
HS3ST4	4,713	9E-32
SCN4B	3,542	9E-32
CLDN19	7,403	2E-31
TRPM3	5,573	2E-31

KLHL13	4,545	2E-31
CHST9	4,397	2E-31
C11orf92	3,695	2E-31
SLC7A14	5,175	3E-31
SYN2	4,030	3E-31
KLHL29	3,808	3E-31
HOXA2	3,503	3E-31
HOXA6	3,525	5E-31
EDN3	6,375	6E-31
FAT2	4,178	6E-31
GRIA4	6,665	7E-31
TP63	4,606	7E-31
COL17A1	5,146	1E-30
KCNJ16	4,658	1E-30
EFCAB1	4,526	1E-30
CLDN11	3,928	1E-30
KCNH8	3,927	2E-30
GAS2	2,920	2E-30
PDE1C	5,070	3E-30
SYNM	4,756	3E-30
HOXA5	3,556	4E-30
SLC7A3	5,988	6E-30
DACT2	4,551	6E-30
TCEAL2	2,910	7E-30
PACRG	2,893	7E-30
CLCA4	5,428	8E-30
GFAP	4,959	8E-30
DRD2	3,522	9E-30
LRRC3B	6,120	1E-29
SCN3A	5,603	1E-29
TSLP	4,412	1E-29
NGFR	3,525	1E-29
GABRA4	14,121	3E-29
EPHB1	4,027	3E-29
SPRY2	3,117	3E-29
PAK3	4,296	4E-29
PSD2	4,212	4E-29
SLC27A6	4,194	4E-29
RNASE7	4,189	4E-29
FEZF2	3,663	4E-29

CCDC8	3,379	7E-29
LOC286367	2,774	7E-29
HPSE2	5,260	8E-29
PTN	4,992	8E-29
ANGPTL7	8,224	1E-28
VIT	4,973	1E-28
SCN5A	3,559	1E-28
RELN	6,192	2E-28
HAP1	4,574	2E-28
NRG2	4,010	2E-28
DST	3,467	2E-28
HOXA4	3,314	2E-28
GPM6B	3,283	2E-28
GPRASP1	3,013	2E-28
EDAR	3,008	2E-28
BOC	2,613	2E-28
LOC728264	6,131	3E-28
WIF1	3,501	3E-28
NRXN1	2,987	3E-28
SPHKAP	5,061	4E-28
DMD	4,337	4E-28
NEUROG2	3,885	6E-28
GNAL	3,106	6E-28
IL11RA	2,231	6E-28
USP44	3,192	8E-28
RIMS3	2,619	8E-28
AQP4	2,638	9E-28
NTF4	4,563	1E-27
TSHZ2	3,770	1E-27
CACHD1	2,875	1E-27
SOBP	2,213	1E-27
SFRP1	4,351	2E-27
CAPN11	3,909	2E-27
RERGL	3,905	2E-27
TEPP	3,817	2E-27
RNF39	2,879	2E-27
KCTD4	2,129	2E-27
KY	5,803	3E-27
SCN2B	4,688	3E-27
RYR3	4,387	3E-27

CLDN8	3,376	3E-27
NDRG2	3,196	3E-27
GRAMD3	2,508	3E-27
TP53AIP1	4,154	4E-27
ASXL3	2,894	4E-27
NPY2R	7,609	5E-27
IL17B	4,741	5E-27
DSC1	4,531	6E-27
C10orf90	3,728	6E-27
HTR2A	3,656	6E-27
SCN3B	2,817	6E-27
ALX4	4,362	7E-27
FAM126A	2,827	7E-27
MRAP2	2,554	7E-27
MRGPRX3	3,939	8E-27
HLF	4,137	9E-27
MATN2	3,482	9E-27
BAI3	2,195	9E-27
FAM150B	4,631	1E-26
ITPRIPL1	3,430	1E-26
WDR86	3,311	1E-26
TRIM29	3,029	1E-26
TCEAL7	3,004	1E-26
TMEM220	2,741	1E-26
HOXA7	2,528	1E-26
NKPD1	2,182	1E-26
KIT	5,198	2E-26
EGFR	4,124	2E-26
LAMB3	2,716	2E-26
EMILIN3	2,597	2E-26
SMYD1	7,093	3E-26
MYH11	4,855	3E-26
STAC2	3,924	3E-26
ID4	3,090	3E-26
HOXA3	2,705	3E-26
PELI2	2,485	3E-26
ZC3H12B	2,309	3E-26
AMOTL1	2,136	3E-26
FAM181A	2,076	3E-26
SCN2A	4,225	4E-26

PTCH2	2,686	4E-26
YPEL4	2,534	4E-26
CNTNAP3	3,689	5E-26
PTPRZ1	2,765	5E-26
SLC25A27	3,189	7E-26
TNS4	2,872	7E-26
OXTR	7,824	8E-26
IRX1	3,389	8E-26
FLJ13197	2,520	8E-26
KCNE1	5,746	1E-25
SLCO1A2	5,067	1E-25
ITM2A	3,059	1E-25
CYYR1	2,341	1E-25
NIPSNAP3B	2,337	1E-25
PTCH1	2,293	1E-25
NCRNA00087	2,256	1E-25
CDKN1C	2,232	1E-25
DPP6	5,321	2E-25
MAMDC2	4,113	2E-25
OR2L13	3,580	2E-25
SOX10	3,549	2E-25
CCL28	3,534	2E-25
AVPR2	2,836	2E-25
FAM13C	2,803	2E-25
DKFZP434L187	2,633	2E-25
AQP1	2,542	2E-25
AASS	2,197	2E-25
ROPN1B	2,013	2E-25
STAB2	4,848	3E-25
ABCA10	4,716	3E-25
SCARA5	4,653	3E-25
PHYHIPL	4,206	3E-25
FAM189A2	3,397	3E-25
CDH12	3,157	3E-25
BBOX1	3,117	3E-25
SH3BGRL2	2,335	3E-25
GNG7	2,252	3E-25
TLE4	2,109	3E-25
DDX26B	2,056	3E-25
PAMR1	4,947	4E-25

ATOH8	3,283	4E-25
SNCA	2,516	4E-25
C1orf190	2,263	4E-25
BEND5	2,099	4E-25
BMP5	2,081	4E-25
EVC2	2,034	4E-25
OSR1	3,716	5E-25
SLC16A12	3,337	5E-25
CGB7	2,971	5E-25
C7orf51	2,519	5E-25
EFNB3	2,369	5E-25
N4BP2L1	2,056	5E-25
CX3CL1	3,891	6E-25
ROBO3	2,579	6E-25
FXYD1	4,105	7E-25
CSRNP3	3,931	7E-25
C21orf34	3,190	7E-25
SOSTDC1	3,823	8E-25
PLD5	3,640	8E-25
CRB2	3,446	8E-25
C18orf34	3,233	8E-25
C11orf93	3,190	9E-25
GLRA4	3,865	1E-24
KCNH3	3,577	1E-24
ASTN1	3,357	1E-24
ABCB1	3,327	1E-24
GIPC2	3,166	1E-24
KLF15	3,088	1E-24
MYOM1	3,062	1E-24
ARL4A	2,609	1E-24
NPM2	2,573	1E-24
DLGAP2	2,563	1E-24
PROS1	2,554	1E-24
PCDHGB7	2,377	1E-24
BACH2	2,212	1E-24
ST8SIA2	4,143	2E-24
DBC1	3,760	2E-24
IL33	3,410	2E-24
TESC	3,385	2E-24
LPHN3	3,183	2E-24

LEPR	3,029	2E-24
NR3C2	2,762	2E-24
CCDC129	2,570	2E-24
IL17RD	2,564	2E-24
ZNF204P	2,447	2E-24
C5orf4	2,240	2E-24
LOC93432	2,101	2E-24
KCNMB1	3,883	3E-24
AK5	3,636	3E-24
SSTR1	2,634	3E-24
FZD7	2,228	3E-24
DCT	5,805	4E-24
CRTAC1	2,457	4E-24
LOC285830	2,091	4E-24
CHST3	2,086	4E-24
TRPC6	2,043	4E-24
NTRK2	3,083	5E-24
RASSF6	2,400	5E-24
TINAGL1	2,290	5E-24
PRIMA1	2,495	6E-24
GPR75	2,250	6E-24
CCDC136	2,243	6E-24
CRHR1	2,139	6E-24
FMO2	3,800	7E-24
MESTIT1	2,907	7E-24
PPP1R12B	2,557	7E-24
SH3TC2	2,429	7E-24
SDPR	3,960	8E-24
ADAMTS5	3,528	8E-24
TSPAN7	2,768	8E-24
SEMA5A	2,446	8E-24
DSC3	2,165	8E-24
TMEM100	2,820	9E-24
TMEM178	3,866	1E-23
C6orf174	2,876	1E-23
ODZ2	2,744	1E-23
LGR6	2,615	1E-23
TDRD10	2,484	1E-23
OR7C1	2,380	1E-23
C9orf125	2,348	1E-23

PRCD	2,187	1E-23
MET	2,052	1E-23
NRG1	3,867	2E-23
ISM1	3,191	2E-23
KRT14	3,110	2E-23
LRRC2	3,096	2E-23
RIC3	2,921	2E-23
EFHA2	2,792	2E-23
PPAP2B	2,110	2E-23
CDC14B	2,078	2E-23
BEGAIN	2,015	2E-23
IGSF10	3,984	3E-23
TACR1	3,639	3E-23
LAMA3	2,588	3E-23
PPP2R2B	2,558	3E-23
SLC35F3	2,413	3E-23
NCRNA00092	2,390	3E-23
PRKD1	2,102	3E-23
RIPK4	2,020	3E-23
CD300LG	4,235	4E-23
LRRTM2	3,228	4E-23
NFIB	2,436	4E-23
TTYH1	2,026	4E-23
TNXB	3,589	5E-23
CPA1	3,330	5E-23
C2orf82	2,880	5E-23
IFFO2	2,353	5E-23
HOXA9	2,322	5E-23
PDGFA	2,303	5E-23
CBX7	2,133	5E-23
LRIG3	2,122	5E-23
CXCL2	5,513	6E-23
AKAP6	2,546	6E-23
LOC441869	2,065	6E-23
MASP1	3,328	7E-23
KCNB1	2,605	7E-23
BMX	3,272	8E-23
GRIK5	2,388	8E-23
NAV2	2,318	8E-23
FGF17	2,254	8E-23

SEMA6D	2,114	8E-23
LOC134466	2,994	9E-23
SLC17A7	2,904	9E-23
FAM13A	2,361	9E-23
LMOD1	3,435	1E-22
C2orf88	3,380	1E-22
CHRDL1	3,359	1E-22
SMAD9	2,446	1E-22
CORO2B	2,360	1E-22
MEIS2	2,311	1E-22
ITGA10	2,278	1E-22
RHOJ	2,188	1E-22
ANKRD35	2,150	1E-22
GSN	2,143	1E-22
MASP2	2,110	1E-22
LPCAT2	2,026	1E-22
CA4	3,760	2E-22
CHL1	3,573	2E-22
GPIHBP1	3,203	2E-22
HAPLN4	2,955	2E-22
KRT5	2,789	2E-22
MAML2	2,747	2E-22
TRPM6	2,393	2E-22
DLG2	2,353	2E-22
SHE	2,199	2E-22
ARHGAP20	3,232	3E-22
MYLK	3,172	3E-22
ALDH1A2	2,941	3E-22
PGM5	2,787	3E-22
FGF2	2,745	3E-22
THSD7B	2,680	3E-22
SYT15	2,313	3E-22
APBA1	2,005	3E-22
CRIM1	2,004	3E-22
FREM1	3,323	4E-22
PLCH2	2,601	4E-22
LOC642587	2,588	4E-22
RGN	2,507	4E-22
LEPREL1	2,235	4E-22
TXNIP	2,094	4E-22

TPO	3,144	5E-22
FGF1	2,923	6E-22
RASSF9	2,609	6E-22
MYOM3	2,569	6E-22
B3GAT1	2,303	6E-22
RASGEF1C	2,143	6E-22
CA3	2,021	6E-22
ABCB5	5,205	7E-22
KCTD14	2,782	7E-22
LOC572558	2,758	7E-22
PDZD4	2,348	7E-22
COL25A1	2,149	7E-22
C7orf41	2,013	7E-22
PDE2A	2,929	8E-22
LGI4	2,230	8E-22
FAM3D	2,825	9E-22
MEOX2	2,587	9E-22
DENND2A	2,427	9E-22
COL4A6	2,226	9E-22
FIGF	5,664	1E-21
ADCYAP1R1	3,488	1E-21
FAM70A	3,072	1E-21
KRT15	2,989	1E-21
ITIH5	2,893	1E-21
KCNA6	2,808	1E-21
PRRG3	2,795	1E-21
JPH4	2,609	1E-21
LOC100126784	2,577	1E-21
TDRD6	2,540	1E-21
PALMD	2,447	1E-21
PHYHIP	2,421	1E-21
LOC399959	2,375	1E-21
GPR146	2,345	1E-21
LCN10	2,298	1E-21
C1orf130	2,296	1E-21
JAM2	2,264	1E-21
TMTC1	2,249	1E-21
TUBB2B	2,233	1E-21
TF	2,231	1E-21
IFNE	2,160	1E-21

ATP1B2	2,097	1E-21
MME	3,892	2E-21
HIF3A	3,593	2E-21
ABCA9	3,524	2E-21
EGR1	3,455	2E-21
KCNMB2	3,377	2E-21
SLC13A2	2,802	2E-21
LOC284578	2,592	2E-21
ANKRD29	2,453	2E-21
TNS1	2,409	2E-21
LOC100124692	2,370	2E-21
IL34	2,322	2E-21
C15orf51	2,263	2E-21
SH3RF2	2,205	2E-21
STOX2	2,175	2E-21
PLSCR4	2,130	2E-21
LTBP4	2,099	2E-21
ARRDC3	2,096	2E-21
ZDHHC15	2,087	2E-21
GLT25D2	2,048	2E-21
PIK3R1	2,046	2E-21
ALDH1L1	2,796	3E-21
KCNS1	2,684	3E-21
RBMS3	2,515	3E-21
KIAA1683	2,354	3E-21
NAP1L2	2,314	3E-21
ART5	2,239	3E-21
TRIM2	2,180	3E-21
BTNL9	3,398	4E-21
PCYT1B	2,319	4E-21
RARB	2,130	4E-21
CYP39A1	2,127	4E-21
SPARCL1	2,109	4E-21
DTX1	2,055	4E-21
CCL14	3,176	5E-21
PTCHD1	2,962	5E-21
GRRP1	2,295	5E-21
MAB21L1	3,169	6E-21
PLCXD3	2,949	6E-21
ITGA7	2,504	6E-21

LAMA1	2,494	6E-21
MOBKL2B	2,130	6E-21
HYMAI	2,078	6E-21
ADRB2	2,568	7E-21
TGFBR3	2,552	7E-21
ANKK1	2,528	7E-21
NKAPL	2,397	7E-21
GLB1L3	2,089	7E-21
PROL1	5,089	8E-21
LIFR	2,842	8E-21
LHFP	2,179	8E-21
CNN1	4,853	9E-21
C4orf49	3,246	9E-21
ACSM3	2,886	9E-21
CSRP1	2,044	9E-21
PIGR	4,270	1E-20
LOXL4	2,931	1E-20
INMT	2,639	1E-20
FOLR1	2,461	1E-20
GGTA1	2,358	1E-20
SCN11A	2,244	1E-20
SLC16A11	2,231	1E-20
PDLIM4	2,199	1E-20
WSCD1	2,180	1E-20
CXorf36	2,177	1E-20
TAL1	2,159	1E-20
ZNF677	2,144	1E-20
IL22RA1	2,027	1E-20
HAS3	4,224	2E-20
ANGPT4	2,926	2E-20
SYT8	2,830	2E-20
PROX1	2,790	2E-20
DNASE1L3	2,689	2E-20
CLDN5	2,558	2E-20
RCAN1	2,557	2E-20
MEOX1	2,537	2E-20
S100B	2,520	2E-20
GSTM5	2,474	2E-20
CADM3	2,459	2E-20
SEMA3G	2,273	2E-20

ZNF483	2,117	2E-20
PIP5K1B	2,093	2E-20
MCF2L2	2,002	2E-20
EGR2	3,198	3E-20
PRDM16	2,643	3E-20
LRRTM4	2,530	3E-20
KL	2,441	3E-20
TGFBR2	2,195	3E-20
CDH23	2,122	3E-20
HEPACAM	3,299	4E-20
DCX	3,278	4E-20
NPR1	2,706	4E-20
LAMC3	2,648	4E-20
NPAS4	2,506	4E-20
AGAP11	2,316	4E-20
MID1	2,219	4E-20
RUNDC3B	2,213	4E-20
ARID5A	2,006	4E-20
PDK4	3,023	5E-20
GREM2	2,592	5E-20
LIMS2	2,332	5E-20
PLEKHH2	2,254	5E-20
FHOD3	2,050	5E-20
ABCA8	3,274	6E-20
DCAF12L1	2,519	6E-20
F10	2,507	6E-20
PEAR1	2,320	6E-20
PRX	2,102	6E-20
GRAMD2	2,007	6E-20
SLC22A11	3,123	7E-20
MYBPC1	2,182	7E-20
CNKSR2	2,630	8E-20
C1orf175	2,374	8E-20
STX19	2,145	8E-20
NTRK3	2,050	8E-20
PRNP	2,002	8E-20
ATP13A5	3,080	9E-20
ADAM33	3,378	1E-19
EGR3	3,109	1E-19
DARC	2,749	1E-19

CAV2	2,472	1E-19
FLJ42875	2,426	1E-19
ZSCAN23	2,406	1E-19
SLAIN1	2,221	1E-19
RHOU	2,094	1E-19
IRX4	2,089	1E-19
TMEM88	2,050	1E-19
SCGB3A1	3,460	2E-19
TMEM132C	3,375	2E-19
CDH22	3,289	2E-19
CKMT2	3,087	2E-19
GPR17	3,037	2E-19
GDF10	2,681	2E-19
FAM107A	2,672	2E-19
RNF150	2,456	2E-19
NLGN1	2,450	2E-19
EBF3	2,373	2E-19
PDGFD	2,212	2E-19
MYH3	2,127	2E-19
GABRP	2,047	2E-19
KLHL33	2,037	2E-19
GAL3ST1	2,018	2E-19
ZNF662	2,003	2E-19
ZBTB16	3,114	3E-19
CAV1	2,713	3E-19
NAALAD2	2,585	3E-19
HSPB2	2,407	3E-19
TCEAL5	2,293	3E-19
LDB2	2,161	3E-19
EMCN	2,160	3E-19
FLRT2	2,155	3E-19
PTX3	2,108	3E-19
ZNF462	2,020	3E-19
KRT17	2,761	4E-19
PPP1R14A	2,617	4E-19
CASP12	2,570	4E-19
ZSCAN4	2,518	4E-19
ANKRD53	2,436	4E-19
USHBP1	2,267	4E-19
RRN3P1	2,078	4E-19

CCL16	2,052	4E-19
OBSCN	2,030	4E-19
RND3	2,010	4E-19
LRFN5	2,677	5E-19
HFM1	2,589	5E-19
MYOCD	2,413	5E-19
WASF3	2,205	5E-19
RAPGEF3	2,087	5E-19
TPTE2P1	2,030	5E-19
PYGM	2,233	6E-19
C1QL2	2,200	6E-19
C4BPA	2,049	6E-19
CLIP4	2,016	6E-19
IGFBP6	2,871	7E-19
SCN4A	2,635	8E-19
VSIG2	2,361	8E-19
GPC3	2,234	8E-19
NTN5	2,146	8E-19
PI16	3,960	9E-19
LUZP2	2,861	9E-19
FHL5	2,581	9E-19
APCDD1L	3,745	1E-18
LCN6	2,583	1E-18
CDH4	2,556	1E-18
ENPP2	2,481	1E-18
APCDD1	2,329	1E-18
EDNRB	2,204	1E-18
PLAGL1	2,128	1E-18
CPAMD8	2,116	1E-18
SPRY1	2,065	1E-18
SPOCK3	2,052	1E-18
ABCA5	2,041	1E-18
SAA4	2,469	2E-18
CD164L2	2,379	2E-18
COL4A3	2,365	2E-18
MT1X	2,269	2E-18
CPEB1	2,255	2E-18
GOLGA6L5	2,196	2E-18
ETV5	2,099	2E-18
CYTL1	2,096	2E-18

CNGA1	2,040	2E-18
TMEM71	2,022	2E-18
RGS7BP	2,021	2E-18
NACAD	2,008	2E-18
SVEP1	2,926	3E-18
CRYAB	2,496	3E-18
LRRC70	2,327	3E-18
CASQ2	2,304	3E-18
OXGR1	2,205	3E-18
ZNF154	2,184	3E-18
S1PR1	2,002	3E-18
NOS1	3,279	4E-18
RUNX1T1	2,299	4E-18
L3MBTL4	2,217	4E-18
CAPN3	2,042	4E-18
JPH2	2,965	5E-18
IGJ	2,763	5E-18
PER1	2,721	5E-18
ABCA6	2,655	5E-18
CNTN1	2,240	5E-18
LRRN4CL	3,040	6E-18
TM4SF18	2,021	6E-18
MMRN1	4,241	7E-18
KCNA1	3,269	7E-18
ALDH1A1	2,471	7E-18
C14orf139	2,381	7E-18
PPYR1	2,223	7E-18
HRNR	2,593	8E-18
ADRA1A	2,531	8E-18
C20orf203	2,300	8E-18
PLA2R1	2,037	8E-18
RIMBP2	2,002	8E-18
MUC15	2,618	9E-18
HSD17B13	3,060	1E-17
ADH1C	2,961	1E-17
RGS2	2,704	1E-17
ACACB	2,507	1E-17
SYNPO2	2,433	1E-17
SOX17	2,391	1E-17
C14orf49	2,139	1E-17

HHATL	6,064	2E-17
ADH4	5,319	2E-17
HEPN1	3,401	2E-17
ATP1A2	3,154	2E-17
CXCL3	2,967	2E-17
ACTG2	2,878	2E-17
SAA1	2,761	2E-17
IGFN1	2,512	2E-17
ACTA2	2,387	2E-17
C10orf128	2,199	2E-17
GLP1R	2,077	2E-17
ETV3L	2,074	2E-17
COL6A6	3,866	3E-17
C1orf61	2,661	3E-17
FAM180B	2,567	3E-17
PAPPA2	2,523	3E-17
MRGPRF	2,283	3E-17
RHOXF1	2,213	3E-17
ENPP6	2,133	3E-17
LY75	2,088	3E-17
UBE2Q2P1	2,086	3E-17
PTPN14	2,054	3E-17
AMPD1	3,047	4E-17
FHL1	2,832	4E-17
SORBS1	2,594	4E-17
SRPX	2,454	4E-17
ANXA1	2,307	4E-17
CCDC158	2,194	4E-17
TNFRSF10D	2,133	4E-17
ECSCR	2,036	4E-17
C1QTNF9	2,555	5E-17
KLF4	2,464	5E-17
FCER1A	2,152	5E-17
ABCG2	2,127	5E-17
ANGPTL1	3,220	6E-17
SLC2A4	3,192	6E-17
CDO1	2,613	6E-17
AMY2B	2,340	6E-17
ZNF727	2,317	6E-17
FAM162B	2,121	6E-17

CABP1	2,060	6E-17
RASL10A	3,579	7E-17
PENK	2,841	7E-17
PPP1R1A	2,615	7E-17
RGS6	2,316	7E-17
P2RY12	2,564	8E-17
ZDHHC8P1	2,014	9E-17
PKHD1L1	5,206	1E-16
NCRNA00110	2,780	1E-16
FAT4	2,073	1E-16
CLEC3B	2,914	2E-16
MFAP4	2,529	2E-16
ADAMTS1	2,468	2E-16
KCNIP1	2,377	2E-16
MST1P9	2,263	2E-16
STK32A	2,254	2E-16
NMUR1	2,247	2E-16
NBLA00301	2,194	2E-16
DBX2	2,119	2E-16
SELP	2,113	2E-16
TMPRSS2	2,055	2E-16
MAP1LC3C	2,927	3E-16
LOC286002	2,462	3E-16
EBF1	2,353	3E-16
BAI1	2,221	3E-16
PIWIL4	2,073	3E-16
MAFF	2,048	3E-16
AQP7P1	2,944	4E-16
PID1	2,300	4E-16
SLC28A3	2,163	4E-16
HSPB6	3,216	5E-16
PLAC9	2,525	5E-16
LOC145820	2,505	5E-16
MEG3	2,399	5E-16
CREB5	2,076	5E-16
GOLGA8A	2,073	5E-16
F3	2,056	5E-16
AMT	2,027	5E-16
MYO1H	2,962	6E-16
RSPO1	2,777	6E-16

RDH5	2,542	6E-16
SAA2	2,430	6E-16
ADH1B	3,111	7E-16
NCAM1	2,636	7E-16
IL1RAPL2	2,577	7E-16
NNAT	2,508	7E-16
SV2B	2,303	7E-16
FAM181B	2,884	8E-16
LRRN3	2,249	8E-16
CLEC4GP1	2,072	8E-16
ANGPT1	2,057	8E-16
SULT1C3	4,074	1E-15
FOSB	4,018	1E-15
ITIH2	3,378	1E-15
ADH1A	3,011	1E-15
CACNA1G	2,444	1E-15
PREX2	2,266	1E-15
RPE65	2,241	1E-15
C1QTNF4	2,234	1E-15
SLC5A1	2,226	1E-15
COL14A1	2,202	1E-15
VIP	2,107	1E-15
GLT1D1	2,094	1E-15
CHRM1	2,067	1E-15
SLC16A7	2,469	2E-15
SCN9A	2,142	2E-15
SOD3	2,106	2E-15
TFPI	2,053	2E-15
RSPO3	2,482	3E-15
PDLIM3	2,251	3E-15
C10orf10	2,119	3E-15
PTGFR	2,096	3E-15
GOLGA8B	2,035	3E-15
FAM55D	2,031	3E-15
ALDH1A3	2,023	3E-15
CCDC69	2,023	3E-15
CCL21	2,906	4E-15
APOB	2,138	4E-15
VWCE	2,018	4E-15
MMP27	2,635	5E-15

ELF5	2,128	5E-15
ABI3BP	2,071	5E-15
CCDC3	2,572	6E-15
NCALD	2,410	6E-15
PAR5	2,307	6E-15
NRN1	2,203	7E-15
LEP	3,311	8E-15
FABP4	2,809	8E-15
CLDN10	3,783	9E-15
GPR182	2,623	1E-14
TP53TG3B	2,580	1E-14
CRHBP	2,459	1E-14
SCN7A	2,399	1E-14
PROKR1	2,371	1E-14
GCOM1	2,288	1E-14
PDZD2	2,126	1E-14
TMC2	2,108	1E-14
ANKRD20A3	2,051	1E-14
SGK1	2,032	1E-14
DMRT2	2,028	1E-14
SLC22A3	2,009	1E-14
AQP7	3,088	2E-14
ACADL	2,540	2E-14
GLRA3	2,417	2E-14
GPR133	2,279	2E-14
NEURL3	2,091	2E-14
PCDHGA3	2,071	2E-14
IQSEC3	2,028	2E-14
ARC	2,016	2E-14
LYVE1	4,847	3E-14
LPL	3,080	3E-14
CD36	2,610	3E-14
AOC3	2,562	3E-14
CCL23	2,457	3E-14
CDH20	2,336	3E-14
PTGS2	2,291	3E-14
KCNIP2	3,092	4E-14
GPX3	2,537	5E-14
HSD17B2	2,194	5E-14
DCDC2	2,179	5E-14

IGF1	2,154	5E-14
LOC283392	3,114	6E-14
GALNTL2	2,404	6E-14
EDN1	2,089	6E-14
CNTFR	2,009	6E-14
CITED1	3,237	7E-14
ASPA	2,501	7E-14
CMA1	3,665	8E-14
CNTN2	2,650	8E-14
SGK2	2,580	8E-14
GLYAT	3,555	9E-14
42433	2,301	9E-14
C7	2,171	9E-14
C14orf180	2,813	1E-13
SLC26A4	2,708	1E-13
TIMP4	2,613	1E-13
ZBTB8B	2,506	1E-13
CSRNP1	2,180	1E-13
CFD	2,085	1E-13
TMEM213	2,056	1E-13
TUSC5	3,184	2E-13
GPD1	3,178	2E-13
CIDEC	3,173	2E-13
GPAM	2,999	2E-13
SEMA3D	2,524	2E-13
ACSM5	2,470	2E-13
HPD	2,260	2E-13
XPNPEP2	2,230	2E-13
DMGDH	2,152	2E-13
PLIN1	3,323	3E-13
ACVR1C	3,260	3E-13
LIPE	2,668	3E-13
ATP13A4	2,009	3E-13
PLIN4	3,082	4E-13
OGN	2,259	4E-13
GPR123	2,083	4E-13
C7orf58	2,050	4E-13
LOC643763	2,239	5E-13
CLEC4G	2,947	6E-13
PPARG	2,353	6E-13

POU3F3	3,010	7E-13
PCOLCE2	2,777	7E-13
MRGPRX2	2,185	7E-13
HSD3B2	2,432	8E-13
SLC19A3	3,122	1E-12
ATF3	2,588	1E-12
AQP7P3	2,341	1E-12
CIDEA	2,300	1E-12
MAOA	2,113	1E-12
LOC90586	2,097	1E-12
AKAP12	2,005	1E-12
MEGF11	3,750	2E-12
HBB	2,983	2E-12
ZFP36	2,328	2E-12
EGF	2,244	2E-12
PCDH8	2,098	2E-12
CXCL12	2,012	2E-12
BTN1A1	2,144	3E-12
FMN2	2,097	3E-12
LRMP	3,212	4E-12
KLB	2,810	4E-12
FRMPD4	2,431	4E-12
C3orf55	2,234	4E-12
ABCD2	2,507	5E-12
EBF2	2,032	5E-12
ZP2	3,880	6E-12
FOS	2,539	6E-12
ADIPOQ	2,917	7E-12
PCK1	2,651	7E-12
C12orf53	2,492	7E-12
DPT	2,467	7E-12
KLHL31	2,155	7E-12
CYR61	2,147	7E-12
DUSP1	2,047	9E-12
FRMPD2	3,632	1E-11
PGA3	2,177	1E-11
GPR15	2,164	1E-11
GHR	2,011	1E-11
RBP4	3,242	2E-11
CCDC141	2,808	2E-11

ODF3L1	2,259	2E-11
SLC26A3	2,213	2E-11
FOXP2	2,129	2E-11
MRAP	3,325	3E-11
HSPB7	3,118	3E-11
NTF3	2,059	6E-11
GGT8P	2,099	7E-11
PRRT4	2,734	8E-11
SLC7A10	2,720	8E-11
PIRT	6,375	1E-10
CSF3	5,150	1E-10
AQPEP	3,428	1E-10
FAM19A1	3,019	1E-10
MIA2	2,629	1E-10
HBA2	2,554	1E-10
LHCGR	2,064	1E-10
SLIT3	2,013	1E-10
KIAA0408	3,122	2E-10
KLK2	2,850	2E-10
LGALS12	2,600	2E-10
FRMD1	2,232	2E-10
GPR109A	2,118	2E-10
KCNA5	2,085	2E-10
GPR97	2,190	3E-10
CTSG	2,060	3E-10
AKR1C1	2,058	3E-10
LST	4,418	4E-10
SCGN	2,706	4E-10
NR4A1	2,388	4E-10
PKD1L2	2,195	4E-10
CD209	2,266	5E-10
PLXNA4	2,220	5E-10
UGT2B28	2,739	6E-10
SCG3	2,092	9E-10
DMP1	2,932	1E-09
MYEOV	2,172	1E-09
CDH8	2,010	1E-09
GYS2	3,050	2E-09
CES1	2,352	2E-09
EPB42	2,207	2E-09

BHMT2	2,060	2E-09
HBA1	2,656	4E-09
G0S2	2,473	4E-09
AADAC	2,711	5E-09
NRSN1	7,530	7E-09
SEL1L2	2,475	9E-09
MUC7	2,634	1E-08
KRT32	2,221	1E-08
MLXIPL	2,173	1E-08
LHFPL3	4,544	2E-08
FAM101A	2,199	2E-08
TMED6	2,839	4E-08
C12orf39	3,900	5E-08
LOC55908	2,290	5E-08
GYG2	2,360	7E-08
LMX1A	2,207	7E-08
COL19A1	2,099	7E-08
