# Supplementary material for: ΔNp73 regulates the expression of the multidrug-resistance genes ABCB1 and ABCB5 in breast cancer and melanoma cells - a short report
Source: Cell Oncol (Dordr). 2017 Jul 4;40(6):631–8. doi: 10.1007/s13402-017-0340-x (PMC5705756; doi:10.1007/s13402-017-0340-x)
Supplement: Supplementary file 3 — (PDF 176 kb) [file 13402_2017_340_MOESM3_ESM.pdf]

Article Title: ΔNp73 regulates the expression of the multidrug-resistance genes *ABCB1* and *ABCB5* in breast cancer and melanoma cells - a short report
Journal Name: Cellular Oncology
Authors Names: Habib A. M. Sakil1, Marina Stantic1, Johanna Wolfsberger1, Suzanne Egyhazi Brage2, Johan Hansson2, and Margareta T. Wilhelm1*

Affiliation: 1) Karolinska Institutet, Department of Microbiology, Tumor and Cell biology (MTC), 171 77 Stockholm, Sweden
2) Karolinska Institutet, Department of Oncology-Pathology, 171 76 Stockholm, Sweden

*Corresponding Author: Margareta T. Wilhelm
email: Margareta.Wilhelm@ki.se

Supplementary Table 3 KEGG pathway analysis. List of genes and pathways that were enriched in ΔNp73 high expressing breast cancer samples.

Category	Term	Count	%	PValue	Genes	List Total	Pop Hits	Pop Total	Fold Enrichm	Bonferroni	Benjamini	FDR
KEGG_PATHWAY	hsa02010:ABC transporters	9	0,992282249	0,001767633	ABCA8, ABCA10, ABCA9, ABCD2, ABCB1, ABCB5, ABCA6, ABCA5, ABCG2	268	44	5085	3,88102103	0,2152421	0,05879565	2,05807023
KEGG_PATHWAY	hsa00350:Tyrosine metabolism	8	0,882028666	0,007322613	DCT, MAOA, ADH4, ALDH1A3, ADH1C, ADH1B, TPO, ADH1A, AOC3, HPD	268	44	5085	3,44979647	0,63464617	0,13397481	8,27616154
KEGG_PATHWAY	hsa03320:PPAR signaling pathway	11	1,212789416	0,002925265	LPL, CD36, SORBS1, PLIN1, PPARG, SLC27A6, FABP4, AQP7, ACADL, ADIPOQ, PCK1	268	69	5085	3,02482154	0,33058337	0,07713261	3,38483211
KEGG_PATHWAY	hsa04512:ECM-receptor interaction	12	1,323042999	0,004135891	LAMA1, LAMB3, LAMA3, CD36, TNXB, COL6A6, LAMC3, ITGA7, ITGA10, SV2B, RELN, COL4A6	268	84	5085	2,71055437	0,43322452	0,09029236	4,75472253
KEGG_PATHWAY	hsa05218:Melanoma	10	1,102535832	0,011287258	EGFR, PDGFA, FGF17, MET, IGF1, PDGFD, EGF, FGF1, FGF2, PIK3R1	268	71	5085	2,67237755	0,7888418	0,17666611	12,4908419
KEGG_PATHWAY	hsa04610:Complement and coagulation cascades	9	0,992282249	0,026979953	C7, F10, MASP1, MASP2, F3, TFPI, C4BPA, CFD, PROS1	268	69	5085	2,47485399	0,97641233	0,2886845	27,4927324
KEGG_PATHWAY	hsa04060:Cytokine-cytokine receptor interaction	31	3,41786108	3,43E-05	CSF3, IL22RA1, PDGFA, CXCL3, LEPR, CXCL2, CNTFR, CX3CL1, KIT, CXCL12, CCL28, CCL23, IL17B, IFNE, CCL21, TPO, EGF, FIGF, GHR, EGFR, MET, TGFBR2, LIFR, EDAR, CCL16, IL11RA, LEP, TSLP, CCL14, TNFRSF10D, NGFR	268	262	5085	2,24500114	0,00468147	0,00468147	0,0402517
KEGG_PATHWAY	hsa04510:Focal adhesion	23	2,535832415	7,47E-04	EGFR, CAV2, CAV1, TNXB, PDGFA, MET, ITGA10, IGF1, COL4A6, LAMA1, PAK7, LAMB3, LAMA3, COL6A6, LAMC3, PAK3, ITGA7, RELN, PDGFD, EGF, FIGF, PIK3R1, MYLK	268	201	5085	2,17114057	0,09734453	0,03356198	0,87483334
KEGG_PATHWAY	hsa04080:Neuroactive ligand-receptor interaction	28	3,087100331	3,45E-04	AVPR2, DRD2, NPY2R, GLRA3, LEPR, TACR1, ADCYAP1R1, LHCGR, PPYR1, GRIK5, OXTR, EDNRB, S1PR1, NMUR1, GABRP, GHR, GABRA4, GRIA4, PTGFR, LEP, CRHR1, ADRB2, SSTR1, CHRM1, ADRA1A, GLP1R, CTSG, HTR2A	268	256	5085	2,07526819	0,04621629	0,02338149	0,40515348
KEGG_PATHWAY	hsa04514:Cell adhesion molecules (CAMs)	14	1,543550165	0,020399401	CLDN8, SELP, CADM3, CLDN19, CLDN5, NLGN1, CLDN10, NRXN1, CLDN11, CDH4, NCAM1, CNTN2, CNTN1, JAM2	268	132	5085	2,01238128	0,94060926	0,26928751	21,5145842
KEGG_PATHWAY	hsa04020:Calcium signaling pathway	16	1,764057332	0,042239183	EGFR, NOS1, TACR1, LHCGR, OXTR, PTGFR, EDNRB, GNAL, ADRB2, PDE1C, RYR3, CHRM1, CACNA1G, ADRA1A, MYLK, HTR2A	268	176	5085	1,72489824	0,99729468	0,38903242	39,7866964
KEGG_PATHWAY	hsa05200:Pathways in cancer	27	2,976846748	0,021392058	PTGS2, PDGFA, FGF17, PPARG, KIT, ZBTB16, ACVR1C, FOS, LAMB3, RARB, EGF, FGF1, FIGF, FGF2, PIK3R1, EGFR, TGFBR2, MET, RUNX1T1, IGF1, FZD7, COL4A6, LAMA1, LAMA3, LAMC3, PTCH1, PTCH2	268	328	5085	1,56187432	0,94831116	0,25639949	22,4443341
