# Supplementary material for: ΔNp73 regulates the expression of the multidrug-resistance genes ABCB1 and ABCB5 in breast cancer and melanoma cells - a short report
Source: Cell Oncol (Dordr). 2017 Jul 4;40(6):631–8. doi: 10.1007/s13402-017-0340-x (PMC5705756; doi:10.1007/s13402-017-0340-x)
Supplement: Supplementary file 4 — (PDF 570 kb) [file 13402_2017_340_MOESM4_ESM.pdf]

Sakil et al.

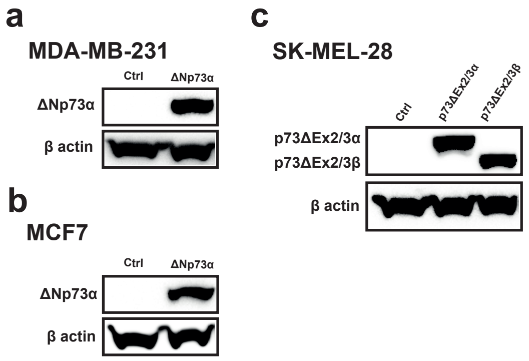

Supplementary Fig. 1 Representative western blots showing overexpression of ΔNp73α, p73ΔEx2/3α and p73ΔEx2/3β isoforms. (**a, b**) Ectopic expression of pcDNA3.1 or pcDNA3.1-ΔNp73α in MCF7 and MDA-MB-231 cells. (**c**) Ectopic expression of pcDNA3.1 or pcDNA3.1-p73ΔEx2/3α and p73ΔEx2/3β in SK-MEL-28 cell lines. Cells were harvested 16 hours after transfection and lysed. Twenty μg total protein lysate was fractionated by electrophoresis and transferred onto nitrocellulose membranes and blotted for p73-isoforms. β-actin was used as a loading control. Representative blots from three independent experiments.
