# Supplementary material for: ΔNp73 regulates the expression of the multidrug-resistance genes ABCB1 and ABCB5 in breast cancer and melanoma cells - a short report
Source: Cell Oncol (Dordr). 2017 Jul 4;40(6):631–8. doi: 10.1007/s13402-017-0340-x (PMC5705756; doi:10.1007/s13402-017-0340-x)
Supplement: Supplementary file 5 — (PDF 655 kb) [file 13402_2017_340_MOESM5_ESM.pdf]

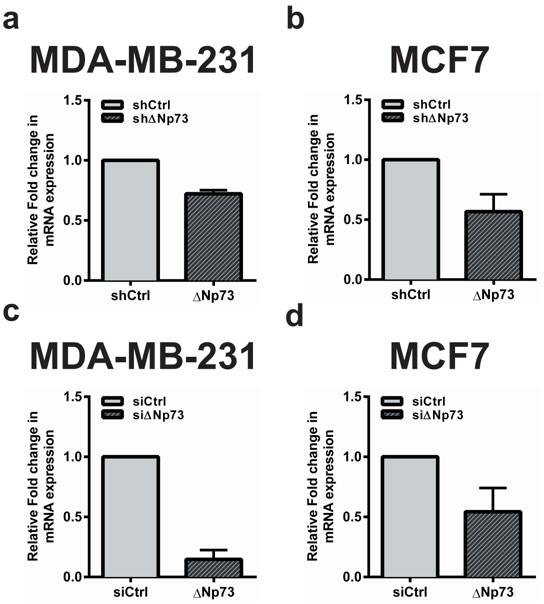

Supplementary Fig. 2 Validation of knockdown efficiency of ΔNp73 in MCF7 and MDA-MB-231 cells. (**a, b**) MCF7 and MDA-MB-231 cells with shRNA-mediated stable knockdown of ΔNp73 in MCF7 and MDA-MB-231 cells. (**c, d**) MCF7 and MDA-MB-231 cells with siRNA-mediated transient knockdown of ΔNp73. Expression levels of ΔNp73 was measured by qRT-PCR using ΔNp73-specific Taqman assays. All samples were run in triplicate in three independent experiments and normalized to GAPDH mRNA. Relative expression was calculated using the ΔΔCT method, and presented as mean fold change±S.E.M.
